# Dental Pulp Stem Cell-Derived Exosomes Alleviate Mice Knee Osteoarthritis by Inhibiting TRPV4-Mediated Osteoclast Activation

**DOI:** 10.3390/ijms24054926

**Published:** 2023-03-03

**Authors:** Yu Fu, Shengjie Cui, Yanheng Zhou, Lixin Qiu

**Affiliations:** 1 Fourth Clinical Division, Peking University School and Hospital of Stomatology, National Center for Stomatology, National Clinical Research Center for Oral Diseases, National Engineering Research Center of Oral Biomaterials and Digital Medical Devices, Beijing 100081, China; 2 Department of Orthodontics, Peking University School and Hospital of Stomatology, National Center for Stomatology, National Clinical Research Center for Oral Diseases, National Engineering Research Center of Oral Biomaterials and Digital Medical Devices, Beijing 100081, China

**Keywords:** osteoarthritis, TRPV4, exosome, osteoclast

## Abstract

Osteoarthritis (OA) is a degenerative disease that causes chronic pain and joint swelling and even disables millions of patients. However, current non-surgical treatment for OA can only relieve pain without obvious cartilage and subchondral bone repair. Mesenchymal stem cell (MSC)-secreted exosomes have promising therapeutic effects on knee OA, but the efficacy of MSC-exosome therapy is not well determined, and the mechanisms involved are still unclear. In this study, we isolated dental pulp stem cell (DPSC)-derived exosomes by ultracentrifugation and determined the therapeutic effects of a single intra-articular injection of DPSC-derived exosomes in a mice knee OA model. The results showed that the DPSC-derived exosomes effectively improved abnormal subchondral bone remodeling, inhibited the occurrence of bone sclerosis and osteophytes, and alleviated cartilage degradation and synovial inflammation in vivo. Moreover, transient receptor potential vanilloid 4 (TRPV4) was activated during the progression of OA. Enhanced TRPV4 activation facilitated osteoclast differentiation, and TRPV4 inhibition blocked this process in vitro. DPSC-derived exosomes repressed osteoclast activation in vivo by inhibiting TRPV4 activation. Our findings demonstrated that a topical, single injection of DPSC-derived exosomes is a potential strategy for knee OA treatment, and that the exosomes regulated osteoclast activation by TRPV4 inhibition, which may act as a promising target for clinical OA treatment.

## 1. Introduction

Osteoarthritis (OA), one of the most prevalent degenerative diseases worldwide [1], manifests itself in chronic pain; joint swelling and stiffness; and finally, disability [2,3]. Alteration of the intra-articular microenvironment, e.g., inflammatory factors and abnormal mechanical stress, stimulates joint resident cells and ultimately initiates degeneration of the articular cartilage and subchondral bone [4,5]. The current treatment strategies mainly focus on pain relief and hardly display structural improvement in the cartilage/subchondral bone. Due to the limited therapeutic effects and unwanted side effects of conservative treatments [6,7], patients with advanced knee OA have to bear the high cost and high risk of joint replacement surgery [8]. Thus, there is an urgent need to seek a new strategy for OA treatment with acceptable cost and excellent tissue repair.

Stem cell-based therapy using mesenchymal stem/stromal cells (MSCs) or their secreted extracellular vesicles, i.e., exosomes, has shown great potential for cartilage regeneration in knee OA [9,10,11]. MSC-derived exosomes can inhibit the overexpression of inflammatory cytokines, induce metabolic reprogramming, and facilitate chondrocytes proliferation [12,13]. However, the mechanism of MSC-derived exosomes for osteoarthritis is not yet completely understood, especially the direct regulation of subchondral bone remodeling. Moreover, an important factor restricting the clinical application of MSC-based therapy is the great difficulty in tissue acquisition, such as bone marrow, umbilical cords, term placentas, etc. Postnatal stem cells in the orofacial system were first identified in 2000 from dental pulp tissue and were named dental pulp stem cells (DPSCs). DPSCs have similar surface markers but stronger colony-forming ability and proliferation ability compared with bone marrow-derived stem cells (BMSCs) [14]. Meanwhile, the extraordinary multilineage differentiation potential [15], the regulation of pericytes through paracrine effect [16], the immunomodulation of the microenvironment [17], and the wide resource of extracted permanent teeth or exfoliated deciduous teeth [18], provide the great possibility of utilizing DPSCs and DPSC-derived exosomes for disease diagnosis [19] and clinical treatment [16,19].

Transient receptor potential vanilloid 4 (TRPV4) is a calcium-related transmembrane channel which can be activated by multiple stimuli, including mechanical stress, osmotic pressure, and bioactive substances [20,21]. TRPV4-activation-induced calcium influx has been shown to promote osteoclast differentiation and regulate bone mass [22]. TRPV4 inhibition has been shown to have therapeutic effects on osteoporosis by inhibiting autophagy through a calcium-dependent pathway [23]. With increasing evidence supporting the idea that TRPV4 plays a vital role in bone metabolism, it may also provide a promising target for subchondral bone loss in OA.

In the present study, we acquired exosomes from human DPSCs and demonstrated the therapeutic effects of a single injection of DPSC-exosomes (DPSC-Exos) on knee OA in vivo. The results showed that an intra-articular injection of DPSC-Exos improved subchondral bone loss, protected the cartilage matrix, and inhibited synovial inflammation. The therapeutic effects were related to TRPV4 inhibition by DPSC-Exos during osteoclast differentiation. Our study identified TRPV4 as a target of DPSC-Exos to improve cartilage and bone degenerative changes and proposes a promising cell-free strategy for knee OA treatment.

## 2. Results

### 2.1. Characterization of DPSC-Derived Exosomes

**Figure 1:**
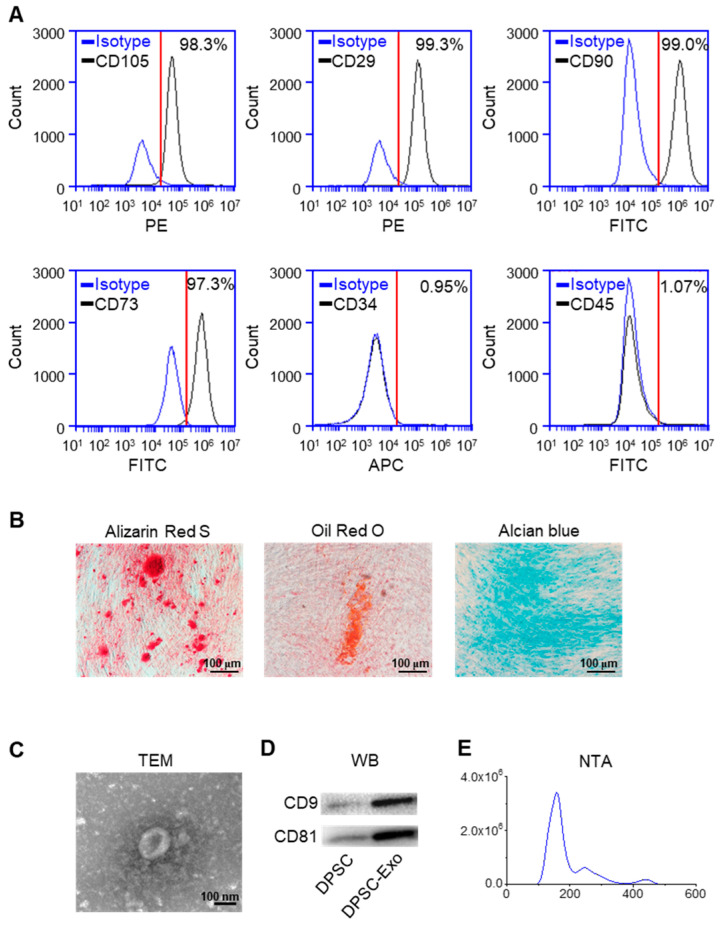
Identification of human DPSCs and exosomes. (**A**) The flow cytometry analysis showed that the DPSCs highly expressed CD105, CD29, CD90, and CD73, while CD34 and CD45 were almost not expressed in the DPSCs. Red line: the threshold of positive cells. (**B**) Alizarin Red S, Oil Red O, and Alcian Blue staining showed that the DPSCs had the capacity for **multi**-lineage differentiation after osteogenic, adipogenic, and chondrogenic induction, respectively. (**C**) Transmission electron microscope image of DPSC-Exos. (**D**) The Western blot results showed high expression of exosome-specific markers CD9 and CD81 in the DPSC-Exos compared with the DPSCs. (**E**) The particle size distribution of DPSC-Exos was analyzed by NanoSight.

The results of flow cytometry showed that DPSCs expressed the markers of MSCs, CD105, CD29, CD90, and CD73 [24,25], and lacked CD34 and CD45, the markers of hematopoietic stem cells (Figure 1A). The results of Alizarin Red S staining, Oil Red O staining, and Alcian Blue staining demonstrated that DPSCs show the potential for multi-lineage differentiation (Figure 1B).

The TEM results showed that the DPSC-secreted extracellular vesicles (EVs) had a round shape with a diameter of ~140 nm (Figure 1C). The results of the Western blot analysis demonstrated a high expression of exosome surface markers CD9 and CD81 in the DPSC-secreted EVs compared with the DPSCs (Figure 1D). The NTA analysis indicated that the diameter of the DPSC-secreted EVs was below 200 nm (Figure 1E). Thus, these results confirmed that the collected DPSC-secreted EVs were exosomes.

### 2.2. DPSC-Exosomes Alleviated Abnormal Subchondral Bone Remodeling of Knee OA

**Figure 2:**
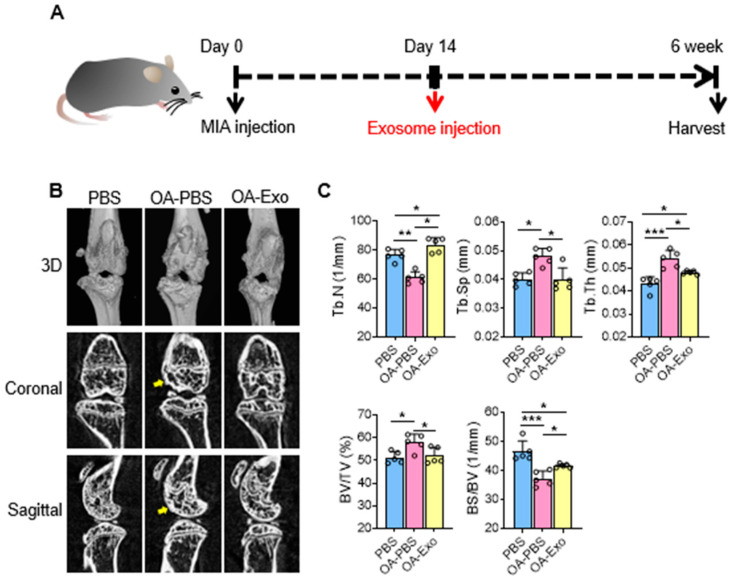
DPSC-Exos local injection effectively relieved bone destruction in progressive knee OA. (**A**) Timeline of induction and treatment of mice knee OA. (**B**) Representative coronal and sagittal view and three-dimensional reconstruction of the knees in the different groups by micro-CT. Yellow arrows: sclerosis or osteophytes. (**C**) Statistical analysis of bone deterioration-relative parameters. Data are presented by mean ± SD. * *p* < 0.05, ** *p* < 0.01, *** *p* < 0.001; *n* = 5.

To evaluate the therapeutic effects of DPSC-Exos, we constructed a mice knee OA model by intra-articular injection of MIA at day 0, and local administration of DSPC-Exos at day 14 (Figure 2A). The results of the micro-CT showed that the MIA injection induced subchondral bone remodeling in both the femur and tibia (Figure 2B). The OA-PBS group displayed abnormal articular morphology by 3D-reconstruction imaging with osteophytes and sclerosis of the cortical bone in the sectional images. MIA injection also resulted in decreased quantities and increased separation of the trabecular bone. In contrast, a single intra-articular injection of DPSC-Exos showed effective protection of the subchondral bone with improvement in cortical bone morphology, alleviation of cortical bone sclerosis, and better preservation of the trabecular bone. The statistical analysis (Figure 2C) showed that the OA-Exo group had significantly increased Tb. N and decreased Tb. Sp compared to the OA-PBS group. The trabecular thickness (Tb. Th) in the OA-PBS group also increased, and it recovered in the OA-Exo group. Furthermore, the results of the BV/TV increased and the BS/BV decreased in the OA-PBS group, and the injection of DPSC-Exos reversed these changes, which was in line with the trabecular bone analysis. Therefore, the results of the micro-CT demonstrated that DPSC-Exos injection effectively alleviated the progression of subchondral bone remodeling in MIA-induced knee OA.

### 2.3. DPSC-Exosomes Improved Cartilage Degradation and Synovial Inflammation of Knee OA

**Figure 3:**
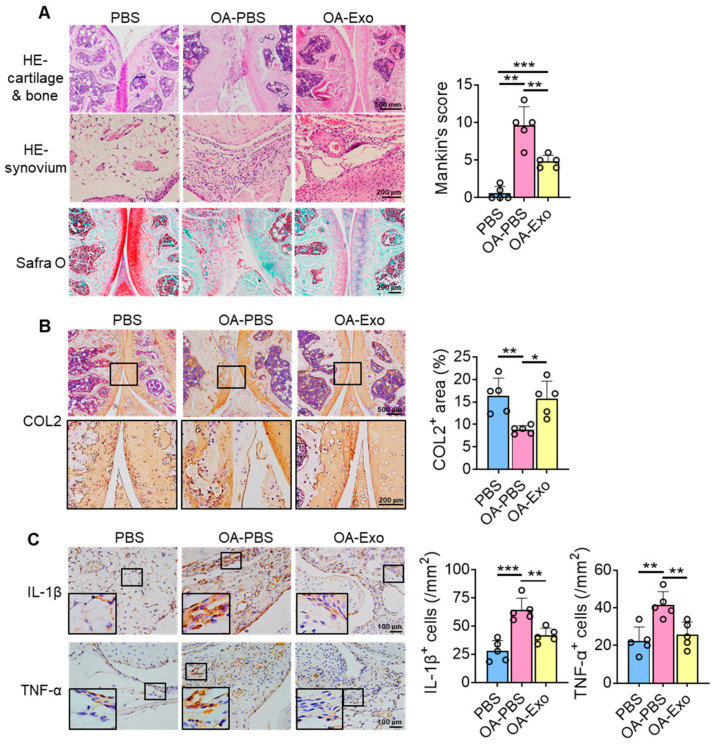
DPSC-Exos local injection improved cartilage degradation and synovial inflammation in progressive knee OA. (**A**) Representative images of HE and Safranin-O staining of the mice knee. The results of Mankin scoring system showed significant difference between OA-PBS and OA-Exo groups. (**B**) Representative images and semi-quantitative analysis of collagen II (COL2) in femoral and tibial cartilage by IHC staining. (**C**) Representative images and semi-quantitative analysis of IL-1β and TNF-α in synovium by IHC staining. The data of semi-quantitative analysis were presented by mean ± SD. * *p* < 0.05, ** *p* < 0.01, *** *p* < 0.001; *n* = 5.

To further investigate the therapeutic effects of DPSC-Exos on knee OA, histological staining and semi-quantitative analysis were performed. The results of HE staining showed that the chondrocytes were well arranged in the cartilage layer, with intact cortical bone below, and that multiple trabeculae were well distributed in the PBS group (Figure 3A). However, the integrity of the joint surface was destroyed in the OA-PBS group, with the cartilage layer being broken, the cortical bone layer thickening, osteophytes being observed, and the trabecular bone decreasing. The OA-Exo group showed an integrated cartilage layer with more trabecular bone compared to the OA-PBS group. Furthermore, the OA-PBS group displayed hyperplasia of the synovial lining layer, with inflammatory infiltration, and the DPSC-Exos injection reduced the quantities of inflammatory cells in the synovial tissue. The results of Safranin-O staining showed that the PBS group had an abundant and integrated cartilage matrix, which was almost diminished in the OA-PBS group and which was partly recovered in the OA-Exo group. The analysis of the Mankin scoring system demonstrated that DPSC-Exos injection significantly improved the pathological changes in the cartilage and subchondral bone of knee OA.

The changes in the cartilage matrix were evaluated by the expression of COL2 by IHC staining (Figure 3B). The results showed that COL2 was highly expressed in the cartilage layer, and that the expression area of COL2 was significantly decreased in the OA-PBS group and partly recovered in the OA-Exo group, which indicated that the DPSC-Exos injection significantly improved the cartilage matrix degradation of OA. Furthermore, changes in inflammatory factors were detected among the different groups. The expression of inflammatory cytokines IL-1β and TNF-α was very limited in the synovial lining tissues in the PBS group, while IL-1β and TNF-α were highly expressed in the OA synovial tissues. The DPSC-Exos local injection significantly inhibited the overexpression of inflammatory cytokines in the synovial tissue (Figure 3C). Taken together, our results suggest that the DPSC-Exos protected the knee joint by regulating cartilage matrix metabolism and inhibiting synovial inflammation.

### 2.4. DPSC-Exosomes Inhibited the Activation of Osteoclasts

Considering the key role of osteoclasts during abnormal subchondral bone remodeling, we applied TRAP staining to evaluate the effects of DPSC-Exos on osteoclast activation in vivo. The results showed that the PBS group had a very limited quantity of TRAP^+^ osteoclasts in the subchondral bone area; meanwhile, the OA-PBS group demonstrated widely distributed osteoclasts with positive staining of TRAP, tightly beside the trabeculae. The OA-Exo group displayed a significantly decreased quantity of TRAP^+^ osteoclasts, which indicated that the DPSC-Exos local injection could partly reverse the activation of osteoclasts.

**Figure 4:**
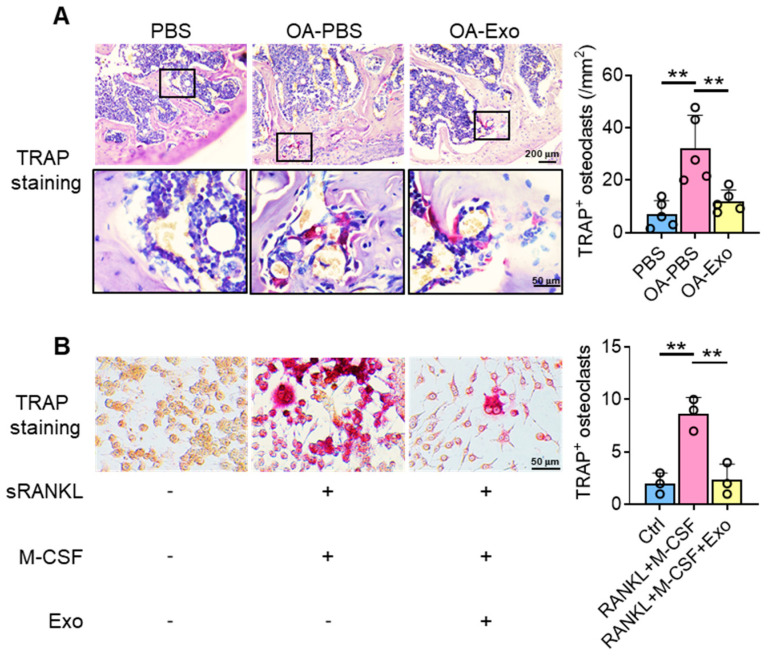
DPSC-Exos inhibited osteoclast activation in the subchondral bone. (**A**) Representative images of osteoclasts in the subchondral bone by TRAP staining. Data are presented by mean ± SD. ** *p* < 0.01; *n* = 5. (**B**) Representative images of RAW 264.7 cells by TRAP staining after osteoclast induction. The data were collected from three independent samples and are presented by mean ± SD. ** *p* < 0.01.

To determine the effects of DPSC-Exos on osteoclast activation, DPSC-Exos was added into the culture medium of RAW 264.7 cells during osteoclast differentiation (Figure 4B). The cultured RAW 264.7 cells were oval/round in shape in the control group, while the cells formed multinucleated giant cells with TRAP^+^ staining after being stimulated with M-CSF and sRANKL. The administration of DPSC-Exos significantly reduced the number of TRAP^+^ cells. Thus, we demonstrated that the therapeutic effects of DPSC-Exos were related to the inhibition of osteoclast activation.

### 2.5. DPSC-Exosomes Regulated the Activation of Osteoclasts via TRPV4

**Figure 5:**
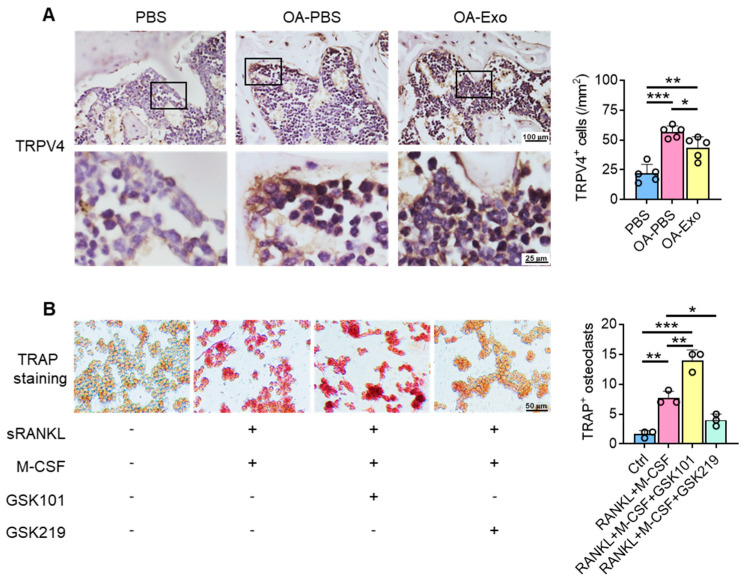
DPSC-Exos regulated osteoclast activation via TRPV4. (**A**) Representative images of TRPV4 in the subchondral bone by IHC staining. Data are presented by mean ± SD. * *p* < 0.05, ** *p* < 0.01, *** *p* < 0.001; *n* = 5. (**B**) Representative images of RAW 264.7 cells by TRAP staining after TRPV4 activation/inhibition. The data were collected from three independent samples and are presented by mean ± SD. * *p* < 0.05, ** *p* < 0.01, *** *p* < 0.001.

To explore the possible mechanisms of the inhibition of osteoclast activation by DPSC-Exos, the expression of TRPV4 was evaluated in vivo (Figure 5A). The cells adjacent to the bone surface showed significantly upregulated expression of TRPV4 in the OA-PBS group, and the DPSC-Exos partly reversed the upregulation of TRPV4, which indicated that TRPV4 participates in the progression of knee OA and may be the target of DPSC-Exos.

We next determined the role of TRPV4 during osteoclast differentiation in vitro. The specific agonist and antagonist of TRPV4, GSK1016790A (GSK101), and GSK2193874 (GSK219), respectively, were delivered into the culture medium during osteoclast differentiation (Figure 5B), and the results showed that TRPV4 activation promoted osteoclast differentiation with TRAP^+^ staining; in their counterpart, the administration of the TRPV4 antagonist inhibited osteoclast differentiation, which was similar to the DPSC-Exos. Therefore, we propose that the therapeutic effects of DPSC-Exos are closely related to the inhibition of TRPV4-induced osteoclast differentiation.

## 3. Discussion

In the present study, we isolated and identified DPSC-derived exosomes and demonstrated the protective effects of DPSC-Exos on MIA-induced mice knee OA. DPSC-Exos effectively improved abnormal subchondral bone remodeling and alleviated cartilage degradation and synovial inflammation. TRPV4 played an essential role in the regulation of osteoclast differentiation, and DPSC-Exos showed therapeutic effects on knee OA by inhibiting TRPV4 activation.

Advanced or end-stage knee OA causes chronic pain or even disability, and current MSC-based therapy has displayed promising treatment effects in preclinical studies and clinical trials [26,27]. However, the implementation of MSCs or MSC-loaded scaffolds still faces some dilemmas, including the resourcing of MSCs, possible ethical issues, the rejection of allografts, and the low degradation rate of exogenous scaffolds [28]. Unlike previous strategies of implanted-MSC with or without scaffolds, or the multi-injection of exosomes [10,12,29], the application of a single injection of DPSC-Exos in the present study is simple and safe. The therapeutic effects of MSCs are closely related to the regulation of the host immune microenvironment and the promotion of host tissue repair/regeneration [17,30,31]. Therefore, MSC-secreted extracellular vesicles can inherit the therapeutic effects of MSCs and are even superior to living cells due to their convenient storage and lower immunogenicity. 

The progression of knee OA is often accompanied by abnormal resorption and repair of the subchondral bone [32], and the occurrence of bone sclerosis and osteophytes is closely related to the prognosis [33,34]. Previous studies mainly paid attention to the therapeutic effects on cartilage degradation and chondrocyte metabolism, while the present study has demonstrated the direct influence of MSC-exosomes (MSC-Exos) on osteoclast activation. Our results have shown that an MIA injection manifested itself in typical radiographic and pathological changes, and DPSC-Exos effectively regulated the bone metabolism via TRPV4 in a progressive OA model. Meanwhile, Hinata et al. [35] claimed the improvement of OA-related functional loss and pain after intra-articular injection of GSK2193874. Therefore, the topical administration of a TRPV4 inhibitor may provide an alternative to improve OA-related subchondral bone remodeling.

TRPV4 is a mechano-conductive calcium channel that is reported to regulate the perception of chondrocytes to the alteration in matrix hardness in the early stage of knee OA [36]. However, some studies have proposed that TRPV4 activation protected chondrocytes from harmful stimulus [37], and other studies have claimed that TRPV4 mediated chondrocyte apoptosis and aggravated mechanical load-induced OA [38]. Therefore, the function of TRPV4 in cartilage and bone may be related to growth and development [39], and may be flexible in the different stages of OA. Understanding the deep mechanisms of TRPV4 in chondrocytes is crucial to improving the efficacy of MSC-based therapies.

Considering the tight relationship between the therapeutic effects of MSC-Exos and the regulation of the topical microenvironment [17], the improved subchondral bone remodeling by DPSC-Exos may not only originate from direct inhibition of TRPV4-mediated osteoclast activation, but also be attributed to the immune microenvironment, such as the balance of macrophage polarization, which is also regulated by TRPV4 [40]. As the immunoregulatory effects of MSC-Exos are tissue-specific [41], it would be worthwhile to further elucidate whether such differences among different MSC-Exos resulted from the capacity of TRPV4 inhibition. 

Although the regulatory effects of MSC-Exos commonly depend on the contained non-coding RNAs and/or proteins, evidence in the present study cannot verify the mechanisms of DPSC-Exos on TRPV4. In summary, the application of DPSC-Exos provides a promising cell-free therapy for OA treatment, but the underlying mechanisms and the efficacy of MSC-Exos derived from different tissues still need further investigation.

In conclusion, the present study collected DPSC-derived extracellular vesicles which showed the characteristic markers of exosomes. These DPSC-Exos showed significant treatment effects on experimental knee OA by improving abnormal subchondral bone remodeling, alleviating cartilage matrix degradation, and inhibiting synovial inflammation. The therapeutic effects came from TRPV4 inhibition during osteoclast activation by the DPSC-Exos. The present study has demonstrated the function of exosomes from an easy-to-obtain cell resource and has provided an alternative for clinical knee OA treatment.

## 4. Materials and Methods

### 4.1. Isolation, Culture, and Identification of Human DPSCs

Human DPSCs were collected from the extracted premolars or wisdom teeth of healthy donors, and digested in 3 mg/mL of type I collagenase (Thermo Fisher Scientific, Waltham, MA, USA) and 4 mg/mL of Dispase (Roche) at 37 °C for 1 h, as previously described [14]. All the procedures were conducted according to protocols approved by the Ethics Committee of the Peking University School of Stomatology (PKUSSIRB-201311103). After passing a 70 μm cell strainer, the cell suspension was cultured with alpha modification of Eagle’s medium (α-MEM, Hyclone, South Logan, UT, USA) containing 15% fetal bovine serum (FBS, Hyclone, South Logan, UT, USA), 2 mM of L-glutamine (Thermo Fisher Scientific, Waltham, MA, USA), and 100 U/mL of penicillin/streptomycin (Thermo Fisher Scientific, Waltham, MA, USA) at 37 °C with 5% CO_2_. DPSCs at passage 3 were used in the following experiment.

A total of 1 × 10^6^ human DPSCs were incubated with fluorescently conjugated antibodies of PE-CD105 (Biolegend, San Diego, CA, USA), PE-CD29 (BD Pharmingen, San Jose, CA, USA), FITC-CD90 (eBioscience, San Diego, CA, USA), FITC-CD73 (Biolegend, San Diego, CA, USA), APC-CD34 (BD Pharmingen, Franklin Lakes, NJ, USA), and FITC-CD45 (BD Pharmingen, San Jose, CA, USA), or isotype-matched IgG controls at room temperature for 1 h. After passing the nylon mesh screen as the filter to remove cell aggregates, the cells were analyzed with a flow cytometer (BD Accuri C6).

To explore osteogenic differentiation, human DPSCs at passage 3 were cultured with an osteogenic induction medium containing 0.1 mM β-mercaptoethanol, 10 mM β-glycerophosphate, 1 nM dexamethasone, and 50 μg/mL ascorbic acid (Sigma-Aldrich, St. Louis, MO, USA), for 21 days, after achieving 70–80% confluency. Then, the cells were fixed for Alizarin Red S staining (Sigma-Aldrich, St. Louis, MO, USA).

To explore adipogenic potential, human DPSCs at passage 3 were cultured with an adipogenic induction medium containing 2 mM glutamine, 0.5 mM 3-isobutyl-1-methylxanthine (IBMX), 1.7 μM insulin, and 1 μM dexamethasone (Sigma-Aldrich, St. Louis, MO, USA). After 21 days, the cells were fixed and stained with fresh Oil Red O solution (Sigma-Aldrich, St. Louis, MO, USA). 

To investigate chondrogenic differentiation ability, human DPSCs at passage 3 were cultured with a chondrogenic induction medium containing 10 ng/mL TGF-β1, (Peprotech, Cranbury, USA), 1% Insulin-Transferrin-Selenium (Gibco, California, USA), 100 nM dexamethasone, 100 μM ascorbic acid, and 2 mM sodium pyruvate (Sigma-Aldrich, St. Louis, MO, USA). After 21 days of cultivation, the cells were fixed and stained with Alcian Blue staining (Sigma-Aldrich, St. Louis, MO, USA).

### 4.2. Isolation of DPSC-Derived Exosomes

After reaching 80% confluency, human DPSCs at passage 3 were cultured in a medium containing exosome-depleted FBS (the supernatants were collected after sequential centrifugation at 2000× *g* for 10 min, at 10,000× *g* for 40 min, and at 120,000× *g* for 6 h [42]) for another 48 h. The culture supernatants were collected and centrifuged at 300× *g* for 10 min, 3000× *g* for 10 min, 20,000× *g* for 30 min, and 120,000× *g* for 70 min to collect the exosomes, as previously described [43]. Exosomes applied in the present study were freshly isolated without being frozen and thawed. 

### 4.3. Characterization of DPSC-Derived Exosomes

For the transmission electron microscopy (TEM) observation, the exosomes derived from 1 × 10^6^ human DPSCs were suspended in 2.5% glutaraldehyde, loaded onto copper grids, and then examined by TEM at 100 kV (JEOL, Tokyo, Japan).

For Western blot analysis, total proteins of human DPSCs or DPSC-derived exosomes were extracted by a protein extraction kit (RIPA Cocktail; Thermo, Waltham, MA, USA), separated by SDS-PAGE, and transferred onto polyvinylidene difluoride (PVDF) membranes (Millipore, Burlington, MA, USA). After being blocked with 5% bovine serum albumin (BSA) and 0.1% Tween-20 for 1 h, the membranes were incubated with CD9 (sc-9148; Santa Cruz Biotechnology, Santa Cruz, CA, USA) and CD81 (sc-9158; Santa Cruz Biotechnology, Santa Cruz, CA, USA) primary antibodies overnight at 4 °C. The blots were then incubated with HRP-conjugated secondary antibodies for 1 h at room temperature and developed by enhanced chemiluminescence detection. Quantitative analysis was performed with ImageLab software.

For nanoparticle tracking analysis (NTA), the exosomes were diluted uniformly in phosphate-buffered saline (PBS) solution and were further measured by a NanoSight analysis system (Malvern Panalytical, Malvern, England) to analyze the size distribution and concentration.

### 4.4. Knee Osteoarthritis Model

The animal procedures were approved by the Peking University Animal Ethics Committee prior to the initiation of the study (LA2021414). To induce progressive knee OA, a total of fifteen C57BL/6 male mice (seven weeks old, 20–25 g) were purchased from SiPeiFu Biotechnology Co., Ltd, Beijing, China. The mice were anesthetized by 1% pentobarbital sodium (8 mL/kg) intraperitoneal injection, and 0.1 mg of sodium mono-iodoacetate (MIA) dissolved in 5 μL of natural saline (NS) was intra-articularly injected into the left knee joint by a microsyringe according to a previous study [44]. Equivalent NS was injected as a control. The mice were randomly divided into OA-NS, OA-Exo, and NS groups (*n* = 5). Two weeks after MIA administration, 10 μL of PBS containing 2 × 10^8^ exosomes was administrated to the OA-Exo group by intra-articular injection, and the equivalent PBS was injected into the NS and OA-NS groups. Five mice were kept at room temperature in a cage with a 12 h dark and 12 h light cycle, and had free access to food and water. The mice were harvested at 6 weeks after MIA administration for micro-CT and histological staining. To minimize pain or discomfort, all of the mice were sacrificed by pentobarbital sodium overdose.

### 4.5. Micro-CT Examination

All the samples were fixed with 10% formalin and scanned with a SkyScan 1174 micro-computed tomography system (micro-CT, 53 kV and 810 μA, Bruker, Billerica, MA, USA). The images were reconstructed and the structural changes in the knee joints of the mice were analyzed using CTan and CTvox software (Bruker) according to a previous study [30].

### 4.6. Tissue Preparation and Histological Staining

Entire knees were removed, fixed in 4% paraformaldehyde, and demineralized in 10% ethylenediaminetetraacetic acid. The specimens were dehydrated in graded alcohol and xylene, embedded in paraffin, and sectioned to 5 μm thickness. The sections were stained with hematoxylin–eosin (HE) staining and Safranin O and Fast Green staining using a Safranin O and Fast Green FCF stain kit (Solarbio, Beijing, China) according to the manufacturer’s instructions. The semi-quantitative analysis of articular cartilage damage was scored using the Mankin scoring system, as previously described [45], and the observers were blinded to the group allocation.

### 4.7. Immunohistochemistry (IHC) Staining

The sections were blocked with 5% BSA and incubated with primary antibodies against COL2 (1:100, sc-52658; Santa Cruz Biotechnology), TNF-α (1:100, sc-1351; Santa Cruz Biotechnology), IL-1β (1:100, sc-52012; Santa Cruz Biotechnology, Dallas, TX, USA), and TRPV4 (1:200, ab39260; Abcam, Cambridge, UK) at 4 °C overnight. After being washed with PBS twice, the sections were incubated with HRP-conjugated secondary antibodies and the staining was visualized by diaminobenzidine. The sections used for the semi-quantitative analysis were obtained from five mice from each group.

### 4.8. Cell Culture and Stimulation

RAW 264.7 cells were cultured with high-glucose Dulbecco’s modified Eagle’s medium (DMEM, Hyclone, South Logan, UT, USA) containing 10% FBS (Hyclone) and 100 U/mL of penicillin/streptomycin (Thermo Fisher Scientific). A total of 5 × 10^3^/well RAW264.7 cells at passages 3 to 5 were seeded into 24-well plates and induced by M-CSF (Peprotech, Cranbury, NJ, USA) and sRANKL (R&D Systems) at a concentration of 20 ng/mL for osteoclast differentiation. Human DPSC-derived exosomes were administrated with a final concentration of 5 × 10^7^/mL.

To investigate the influence of TRPV4, the RAW 264.7 cells induced by M-CSF and sRANKL were treated with GSK219 (10 μM; Selleck, Houston, TX, USA), a specific TRPV4 inhibitor [46]. TRPV4 agonist GSK101 (Selleck Selleck, Houston, TX, USA) was delivered at a concentration of 100 nM [37]. DMSO was administrated as a control.

### 4.9. Tartrate-Resistant Acid Phosphatase (TRAP) Staining

The sections were deparaffinized and stained with a TRAP stain kit (Solarbio, Beijing, China) according to the manufacturer’s instructions. TRAP-positive multinucleated cells attached to the bone surface were counted (*n* = 5).

The RAW 264.7 cells were fixed and stained with a TRAP stain kit (Solarbio, Beijing, China) according to the manufacturer’s instructions. TRAP-positive multinucleated osteoclasts were counted and analyzed from three independent samples.

### 4.10. Statistical Analysis

The statistical analysis was conducted with GraphPad Prism software (v8.0). After testing the normality of the data by using the Shapiro–Wilk test, all the data were subject to normal distribution and were presented as mean ± SD. Differences observed in histological staining, micro-CT, and other experiments were assessed by one-way ANOVA with the Holm–Sidak test. *p*-values < 0.05 were considered statistically significant.

## Data Availability

Not applicable.
